# Prediction and evaluation of healthy and unhealthy status of COVID-19 patients using wearable device prototype data

**DOI:** 10.1016/j.mex.2022.101618

**Published:** 2022-01-10

**Authors:** Shaik Asif Hussain, Nizar Al Bassam, Amer Zayegh, Sana Al Ghawi

**Affiliations:** Centre for Research and Consultancy, Middle East College, Muscat, Oman

**Keywords:** Quarantine, Wearable electronic device, Pandemic, Healthcare, AI model, Dataset

## Abstract

COVID-19 pandemic seriousness is making the whole world suffer due to inefficient medication and vaccines. The article prediction analysis is carried out with the dataset downloaded from the Application peripheral interface (API) designed explicitly for COVID-19 quarantined patients. The measured data is collected from a wearable device used for quarantined healthy and unhealthy patients. The wearable device provides data of temperature, heart rate, SPO_2_, blood saturation, and blood pressure timely for alerting the medical authorities and providing a better diagnosis and treatment. The dataset contains 1085 patients with eight features representing 490 COVID-19 infected and 595 standard cases. The work considers different parameters, namely heart rate, temperature, SpO_2_, bpm parameters, and health status.

Furthermore, the real-time data collected can predict the health status of patients as infected and non-infected from measured parameters. The collected dataset uses a random forest classifier with linear and polynomial regression to train and validate COVID-19 patient data. The google colab is an Integral development environment inbuilt with python and Jupyter notebook with scikit-learn version 0.22.1 virtually tested on cloud coding tools. The dataset is trained and tested in 80% and 20% ratio for accuracy evaluation and avoid overfitting in the model. This analysis could help medical authorities and governmental agencies of every country respond timely and reduce the contamination of the disease.

• The measured data provide a comprehensive mapping of disease symptoms to predict the health status. They can restrict the virus transmission and take necessary steps to control, mitigate and manage the disease.
• Benefits in scientific research with Artificial Intelligence (AI) to tackle the hurdles in analyzing disease diagnosis.
• The diagnosis results of disease symptoms can identify the severity of the patient to monitor and manage the difficulties for the outbreak caused.

## Graphical abstract

**Figure:**
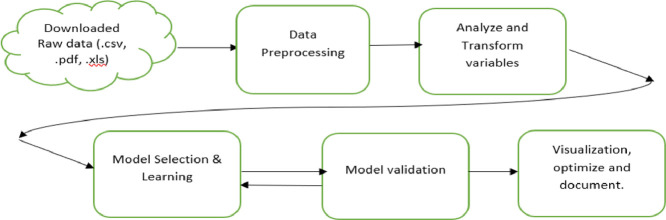

Specifications table

**Table:** 

Subject Area	Engineering
More specific subject area	Data Mining- Artificial Intelligence
Method name	Random Forest Classifier Algorithm used to train and test the data to predict the disease progression
Name and reference of original method	NA
Resource availability	https://doi.org/10.5281/zenodo.4766192 http://www.c19data.info/index.php/admin/patients

## Methodology and data

**Fig. 1:**
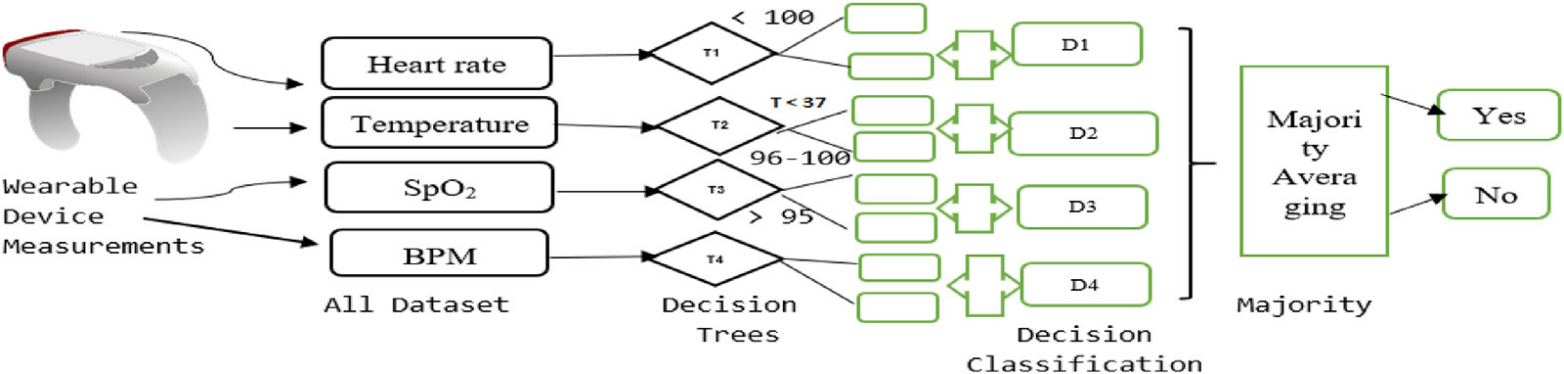
Shows the RF model classification.

The method used for the Data mining classification is Random Forest Algorithm for machine learning. Generic Machine Learning is employed to build a diagnosis model for COVID-19 patient symptoms with the steps involving support vector machine, Decision tree, and Random Forest, and logistic regression for processing the diagnosis data to detect COVID-19 cases (Fig. 3). The random forest algorithm is a classifier built to diagnose the disease from the signs and symptoms of COVID-19 patients [8]. The (Fig. 1) shows the design flow employed to judge the essential and represent an AI project which can build a model to gather every possible data and give us an insight understanding to analyze the health status of COVID-19 patients.

### Data descriptive and statistics

The dataset contains four measured values taken from a wearable device fixed with individual sensors of Temperature, blood pressure, heart rate, and SpO_2_ as given in Table 1. The dataset includes 1085 patients with eight features representing the proportion of balanced data (Table 3). Through the web platform, dataset is downloaded for the patients in .CSV, PDF, and Excel format consist of 8 columns and 1085 rows [12]. The source file is a collection of data from the given API link ProjectC (c19data.info) (Table 4). The proposed work has also been tested and implemented in the Anaconda tool (AEN 4.1 version) for data analysis [1].

We can read the dataset as a supplementary file easily in .CSV forma (Table 4). The data is updated and stored from the above API link is provided. Random forest Algorithm is composed of different decision trees with supervised learning to perform both regression and classification (Fig. 4). The algorithm is a diverse model with decision trees, nodes, and leaves to classify unlabeled data [6]. In the proposed work, numerical data with irrelevant attributes such as Patient Id, gender, age, Heart rate, temperature, SpO_2_ saturation, blood pressure monitor [4]. The informative data values are selected to predict the health status and probability of infection among these attributes [3]. The algorithm shows the sample dataset of COVID-19 patients to associate a set of training documents with the selected features Table 2, Table 3, Table 4, Table 5, Table 6, Table 7, Table 8, Table 9, Table 10. The data classification is carried out with the real-time measurements collected from different patients [13], commonly known as a definite response, to predict the output Y from the input variables X (Table 8). In actuality, the relationship is between response and predictors [4]. The background classification is carried out with nearest neighbors’ classifiers to obtain the linear model classification (Table 6).

This work uses supervised learning with inputs and correct outputs to model the dataset over time to yield the desired outcome from the diagnostic devices to minimize the error sufficiently [10]. The method used to model is Random Forest classifier where scikit- learn version 0.22.1 and python version is 3.7.5 was used and tested on google colab. Multi-class classification gives the best understanding of the measured performance with one part of data as a training set and another for testing data [3]. The following steps explain the performance metric and splitting strategy, where the raw data is converted into a sequence to analyze from a viewpoint (Table 5). The proposed work has also been tested and implemented in the Anaconda tool (AEN 4.1 version) for data analysis [1].

**Table 1:** The parameters in the dataset.

Data parameters	Description	Attributes
Gender	Patient gender is an attribute primary spectrum of Health care	Male or female
Age	Patient's age is major influence associated to determine the health care	Less than 80
Heart Rate	Pulse defines heart beats per minute as either too fast or too slow	< 100
Temperature	Body temperature in human to evaluate person's health	< = 37
SpO_2_ Saturation	It measures the percentage of blood oxygen content and arterial saturation	96–100%
Blood pressure	Measures the blood pressure in the circulatory system	> 95

**Table 2:** Shows the dataset shape for first five rows from the loaded dataset.

S.No.	Patient ID	Gender	Age	Heart_rate	Temperature	SpO_2_ Saturation	BPM	Health_Status
0	1	Male	66.0	70	38.6	88.0	75	Infected
1	2	Female	56.0	74	39.6	88.0	70	Infected
2	3	Male	46.0	82	37.2	98.0	83	Non-Infected
3	4	Female	60.0	90	38.6	98.0	75	Non-Infected
4	5	Male	58.0	72	39.6	93.0	78	Infected

**Table 3:** Data Columns and types with count (total 8 columns).

#	Column	Non-Null count	Dtype
0	Id	1085 non-null	Int 64
1	gender	902 non-null	Object
2	Age	843 non-null	Float 64
3	Heart_rate	1085 non-null	Int 64
4	Temperature	1085 non-null	Float64
5	SPO_2__saturation	1085 non-null	Float64
6	Bpm	1085 non-null	Int 64
7	Health_status	1085 non-null	Object

Dtypes: float64(3), int64(3), object (2); Memory Usage: 67.9+ kB.

**Table 4:** Shows the dataset file with all the data included.

S. No.	id	Gender	Age	Heart_rate	Temperature	SpO_2_ Saturation	bpm	Health_status
0	1	Male	66.0	70	38.6	88.0	75	Infected
1	2	Female	56.0	74	39.6	88.0	70	Infected
2	3	Male	46.0	82	37.2	98.0	83	Non Infected
3	4	Female	60.0	90	38.6	98.0	75	Non Infected
4	5	Male	58.0	72	39.6	93.0	78	Infected
…	…	…	…	…	…	…	…	
1080	1081	NaN	24.0	110	38.0	30.0	72	Infected
1081	1082	NaN	35.0	110	38.0	30.0	74	Infected
1082	1083	Male	NaN	110	38.0	30.0	68	Infected
1083	1084	Male	NaN	110	38.0	30.0	67	Infected
1084	1085	Male	70.0	110	38.0	30.0	70	Infected

**Table 5:** Shows the standard statistics calculated for the considered data.

	id	age	Heart_rate	Temperature	SpO_2_ Saturation	bpm
Count	1085.000000	843.000000	1085.000000	1085.000000	1085.000000	1085.000000
Mean	543.000000	49.483689	89.812903	38.562488	66.707465	71.221198
std	313.356825	18.255334	19.685747	4.592419	30.251069	13.148559
Min	1.000000	0.250000	47.000000	36.000000	20.000000	44.000000
25%	272.000000	35.000000	72.000000	38.000000	30.000000	59.000000
50%	543.000000	51.000000	91.000000	38.100000	82.000000	72.000000
75%	814.000000	64.000000	110.000000	38.500000	87.300000	81.000000
max	1085.000000	96.000000	120.000000	95.000000	340.000000	109.000000

**Table 6:** Shows the correlation coefficient for the dataset.

	id	age	Heart_rate	temperature	SpO_2_ Saturation	bpm
ID	1.000000	−0.033531	0.721335	−0.082765	−0.558897	0.001511
Age	−0.033531	1.000000	0.083925	0.091438	0.033087	0.061741
Heart_rate	0.721335	0.083925	1.000000	−0.028797	−0.235919	0.284245
Temperature	−0.082765	0.091438	−0.028797	1.000000	0.054208	0.003302
SPO_2_ Saturation	−0.558897	0.033087	−0.235919	0.054208	1.000000	0.079131
bpm	0.001511	0.061741	0.284245	0.003302	0.079131	1.000000

**Table 7:** Shows the criterion of parameters for train and test points.

S. No.	Parameters	Infected (Non-Healthy)	Non-Infected (Healthy)
1.	Temperature	*T* > 37	*T* < 37
2.	Heartbeat variation	> 100	< 100
3.	BPM	<= 94	> 95
4.	SpO_2_	95–100%	< 94%

**Table 8:** Dataset to measure Accuracy.

Description	Parameters (X, Y)	Percentage
Accuracy score	Y_test and Y-Predict	0.9926470588235294
Training score	X_Train and Y-Train	0.968019680196802
Testing score	X_train and X-Test	0.9705882352941176

**Table 9:** Training and testing data for randomized values for 813 rows x 4 Columns.

Id:813 rows x 4 Columns	Heart_rate	Temperature	SpO_2__ saturation	bpm
862	113	38.5	30.0	67
658	97	38.5	85.0	66
252	78	36.9	98.0	67
706	102	38.5	85.0	53
215	64	37.8	85.0	81
…	…	…	…	…
1033	110	38.0	30.0	75
763	109	38.5	87.3	82
835	112	38.5	30.0	77
559	70	37.6	30.0	57
684	95	38.5	85.0	94

**Table 10:** Training and testing data for randomized values for 272 rows x 4 columns.

Id: [272 rows x 4 columns]	Heart_rate	Temperature	SpO_2__ saturation	bpm
204	61	38.0	85.0	89
183	65	37.8	89.0	94
356	82	37.1	96.0	58
1069	118	38.0	30.0	86
272	85	38.0	90.0	70
…	…	…	…	…
255	87	38.0	98.0	76
495	57	38.1	30.0	57
319	71	38.1	85.0	74
493	62	38.1	55.0	56
144	77	39.6	82.0	84

#### Pseudo code for RF algorithm

1. From the total ‘K’ features, select the informative attributes as ‘n’ features. Here the condition is *n* << K.
2. Now, for the n features defined calculate the best point for splitting the features.
3. Each node is classified as best split into daughter nodes.
4. Perform the steps from 1 to 3 until the number of nodes reaches 1.
5. Hence the n number of trees are generated to deploy and build the Random Forest model from 1 to 4.

#### Dataset classification

**Fig. 2:**
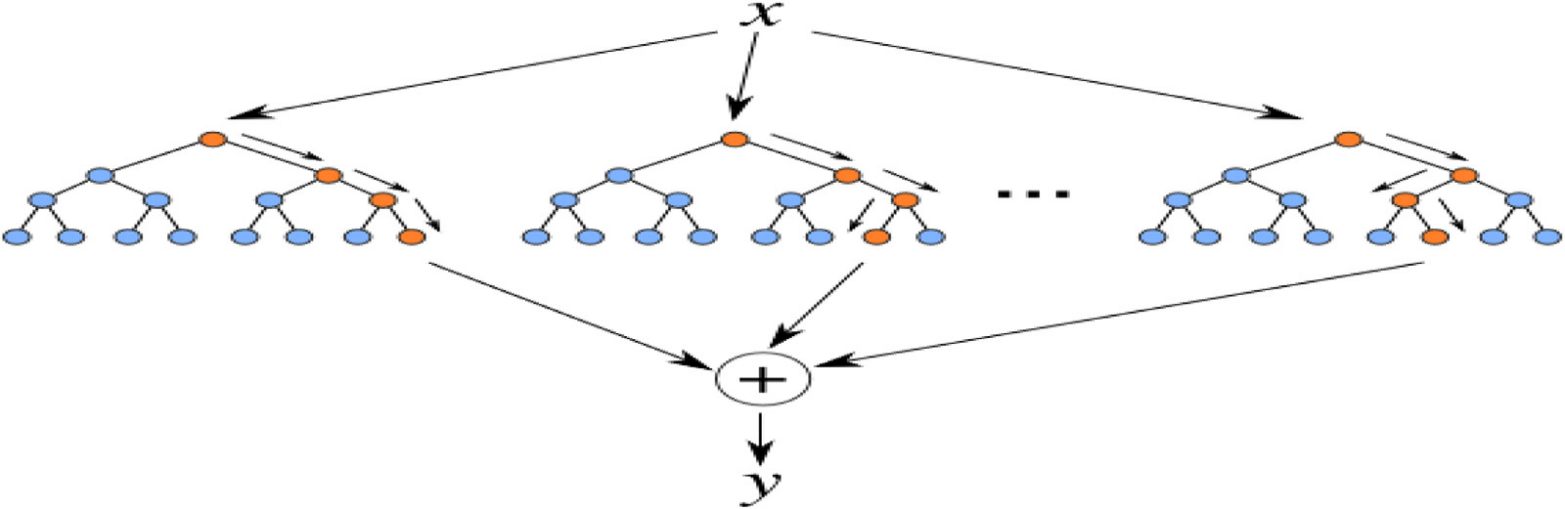
Shows the process of classification with X and Y as actual and predicted values. (https://dsc-spidal.github.io/harp/docs/examples/rf/).

**Fig. 3:**
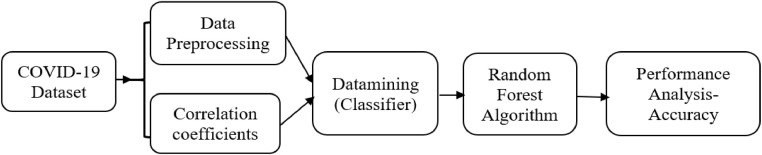
Dataset modeling, classification, and prediction.

**Fig. 4:**
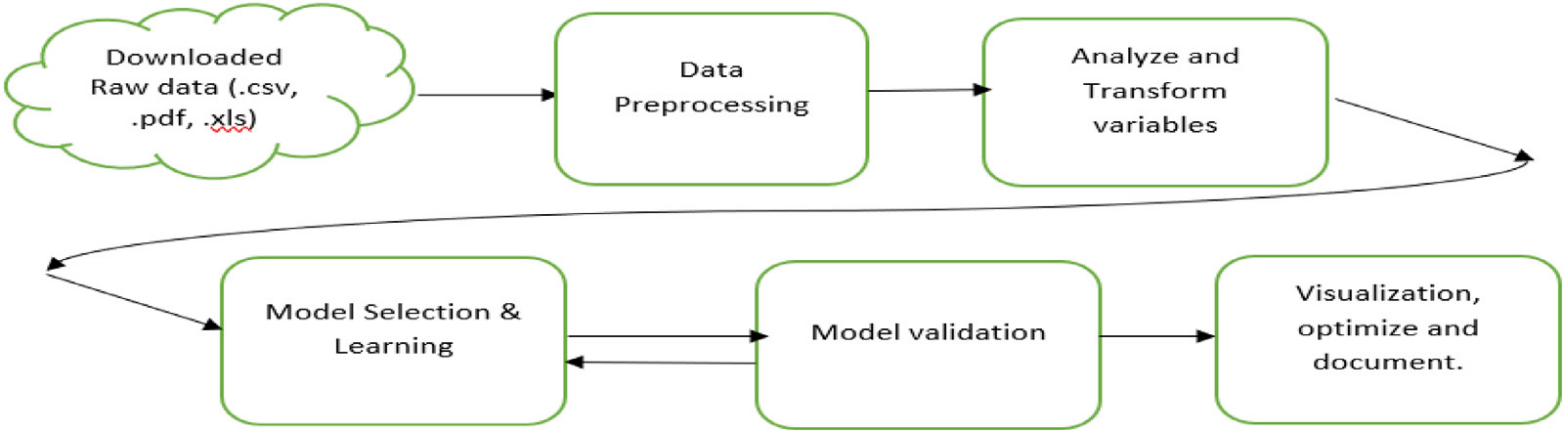
The design flow model of machine learning for COVID-19 dataset.

**Fig. 5:**
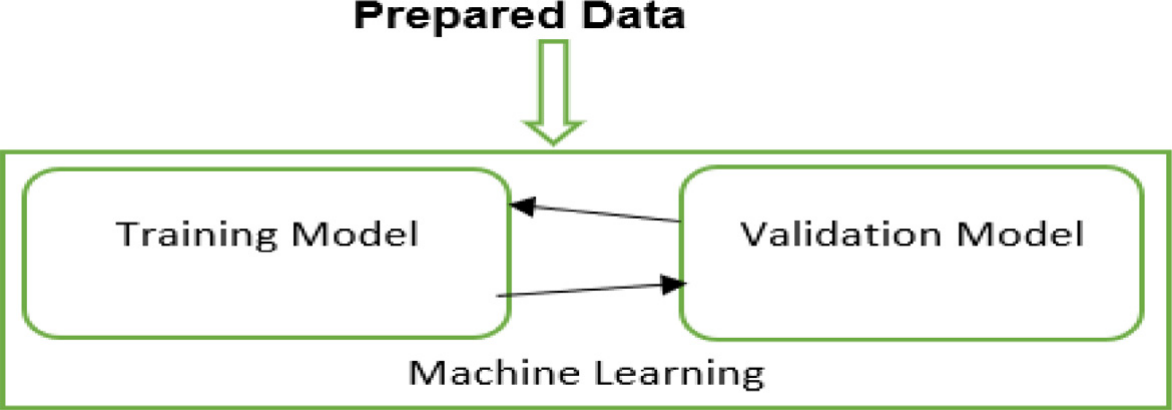
The performance estimation and predictive model flow.

Random Forest algorithm is chosen as the best among the classifiers as it takes very little time for training and overfitting [2]. Also, its significant feature is the level of accuracy to predict class-wise error rate (Figs. 2– 5).

• The tree classification of the RF model to the following steps.
• A binary tree is grown to classify the data.
• Nodes are defined to indicate and separate the data into two as daughter nodes.
• Splitting is done based on the conditions or scaled values.
• End nodes are known as terminal nodes. The prediction of the class is classified based on the majority of trees.
• The splitting criteria are classified based on the Gini criterion or conditions defined.
  $$\text{Gini}=N_{L}\sum_{K=1\ldots K}p_{KL}\left(1-p_{KL}\right)+\;N_{R}\sum_{K=1\ldots K}p_{KR}\left(1-p_{KR}\right)$$
  Gini = $1-\sum_{i=1}^{c}{\left(P_{i}\right)}^{2}$

p_KL_ = Left node in proportion of class K.
p_KR_ = Right node in proportion of class K.

**Fig. 6:**
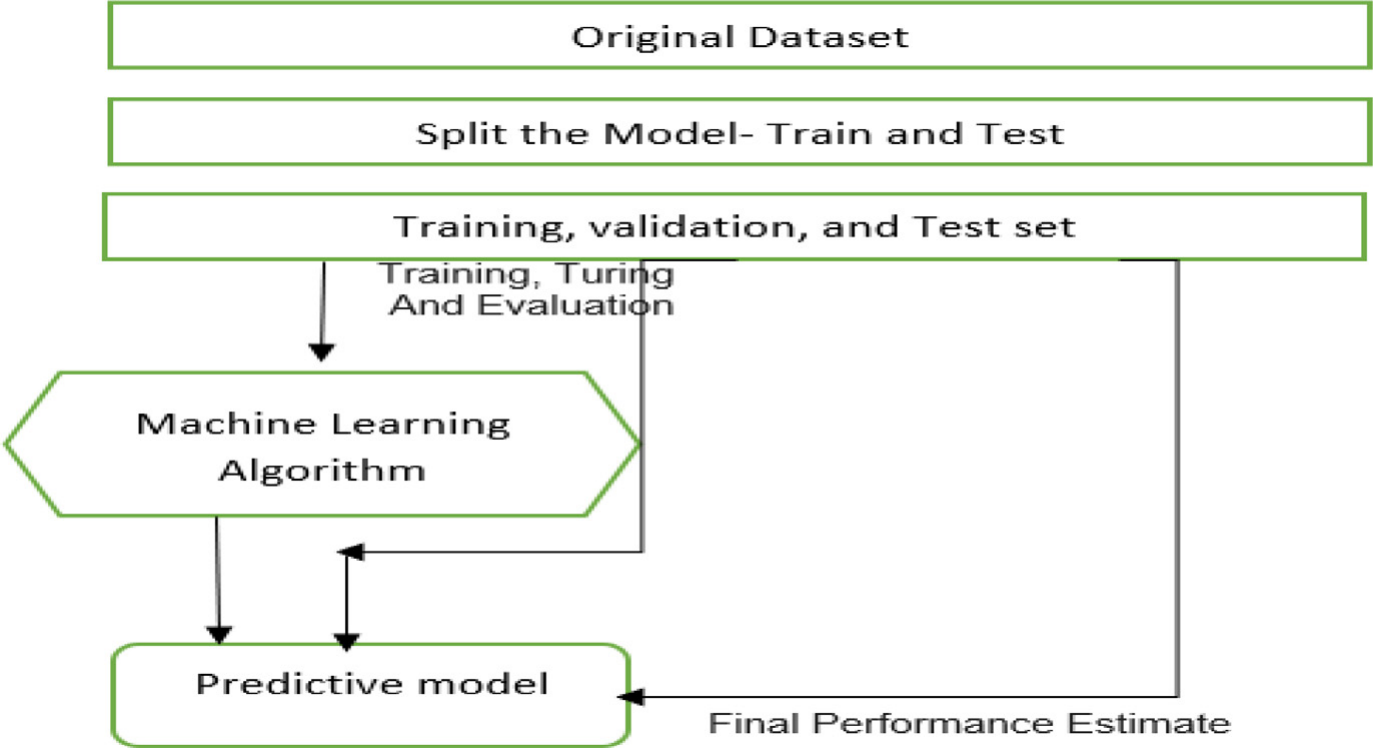
The performance estimation and predictive model flow.

Regression The technique used to estimate the difference from independent feature to dependent features is linear regression which can easily forecast and predict the impact of relationship variables [5].

## Algorithm procedure

A Random Forest algorithm extracts the subsamples from the given dataset to the ensemble datasets (Table 7). The dataset contains eight features, with four features are relevant attributes having a meaningful relationship.

The algorithm works in two phases as random bootstrap sampling and decision trees creation. These methods together are used to classify the result for the prediction. In the first phase, it uses the bootstrap sampling method to bootstrap the samples as f_1_(x), f_2_ (x) ...F_M_(x) to obtain f(x) utilizing model averaging. The second phase defines the criteria in classifying the trees as daughter nodes and implements a simple vote [7].

This work considers a mathematical and AI approach for the real-time dataset of COVID-19 patients to determine the current state of infection from SpO_2_ saturation, temperature, heartbeat, and blood pressure values [9]. The current health state trained and tested from the dataset gives a data-driven model to monitor and forecast the pandemic health condition of different patients [11].

### Illustrative Pseudo code with python programming

# Importing Libraries
Import pandas as pd
# Load dataset from your local drive
DATASET_LOC = /path/downloads/covid-19-26.csv
# Correlates all the attributes
Correlation = correlation.colums
Plt.scatter= Range Index(start=0, stop=1085, step=1)
#InteractiveShell
from IPython.core.interactiveshell
InteractiveShell.ast_node_interactivity = ``all''
# split train and test and fit the model
from sklearn.model_selection
dcf= RandomForestClassifier()
# Creating training and test sets
X_train, X_test, y_train, y_test = train_test_split(X, y, test_size = 0.2, random_state = 0)
#Inference on validation of dataset
Pred= dcf.predict_model
# Accuracy check and stats for inference
accuracy_score(y_test,y_predict)
lr.score(X_train, Y_train, X_test, Y_test)

1. To implement and understand the work carried the following steps are defined.
2. Load the dataset in google colab or visual code (Table 4).
3. Add the proposed work in the Anaconda tool (AEN 4.1 version) for data analysis (Table 5).
4. The dataset is loaded, and it displays the first five rows of the data frame packed in the above software tool used. The command used to display the five rows is df. head (Table 2)

The dataset shape is obtained by using print statement as Dataset Shape: (1085, 8)

## Data wrangling, collection, and cleaning

The raw data can perform meaningful analytics and train a machine learning model. The data stored in .CSV (comma Separated) file format determines the relevant attributes collected for patients of age and gender with symptoms and signs of SPO_2_ saturation, heart rate, blood pressure, and temperature (Fig. 6). The data cleaning step is to remove missing values and unwanted characters used in the data. df in the code indicates drawn data frame and the null values by using autocleaning and summing the predicted null values to perform data manipulation operations.

1. The correlation coefficients represent a relationship between two variables where it is a relationship between dependent and independent variables. The features for each attribute are separately shown in each column to define the variables in the dataset (Table 6). The above step avoids false repetition of the values. The below Eq. 1 represents with ∑a and ∑bfor first and second variable values, m is the quantity information.
  (1) $$r=\frac{m\;(\sum ab)-(\sum a)-(\sum b)}{\sqrt{\left(\left[m\sum a^{2}-{\left(\sum a\right)}^{2}\right]\left[n\sum b^{2}-{\left(\sum b\right)}^{2}\right]\right)}}\ldots \ldots .$$
2. When multiple lines are in a cell, an interactive shell defines the core simulation. In our dataset, relevant features from columns 3–7 are considered, with x defining the input response and y is predicted outputs. The head represents the first five rows of x and y (Table 2). For the dataset based on the conditions, split into train and test. This step maps the data in an optimal format for selecting a training set to process the data together, known as feature transformation.
3. Splitting data into training and testing Sk learns function separates the train and test data from the source dataset by specifying the test size and train size (Table 10).

4. The model is fitted based on the parameters assigned in the random forest model. This model specifies the parameters such as features per node, num Trees, max Tree depth, RF predictor, confusion matrix. It set the best fit model for the random forest classifier. In this step, the algorithm is trained for evaluation to ensure proper testing. The data is split with 80% for training and 20% for testing to refine and optimize the model over time (Table 9).

5. The model is classified with the dataset to measure accuracy by using binary classificatory as the following (Table 8).
  $$Accuracy=\;\frac{TP+TN}{TP+TN+FP+FN}$$

Where True positive (TP), True negative (TN), False positive (FP), and False Negative (FN) are the metrics for non-binary classificatory, the data of machine learning model determines the highest probability as overall accuracy where a correct number of segments are counted as an actual class and divided by the total number of elements.

1. Model validation: The training and testing data are the same, where the data is split into training data to test the final model. The data has classes to define overfitting and underfitting to generalize the data. In this work, overfitting applies to the training data as the value obtained is too close to the outcome (Table 9).
2. To predict the classification and its score, a confusion matrix is used. The matrix information collects actual and predicted information in a separate column specifying the health status Table 2, Table 3, Table 4.

## Conclusion

This simulation study has analyzed the risk of COVID-19 disease progression using random forest classifier algorithm. The eight features intensify the uncertainty to forecast the disease progression, which has brought health and financial crisis. The result has predicted the accuracy score of 99.26%, with training and testing scores separately as required. The 1085 samples used have total volatility to spillover during diversity. The comprehensive open-source framework of google colab uses Anaconda AEN 4.1 version with designed efficiency to parameterize many body functions in artificial neural networks. The random forest classifier algorithm shows the sample dataset of COVID-19 patients to associate a set of training documents with the selected features. The jupyter notebook software offers a real-time simulation with attributes for informative data values, which are determined to predict the health status and probability of infection. The data analysis used is to predict the classification and its score confusion matrix as 96.8 and 97.05%. This performance uses a classification process of two classes in the form of the available data matrix. The matrix information collects actual and predicted information in a separate column specifying the health status.

## CRediT authorship contribution statement

**Shaik Asif Hussain:** Methodology, Software, Data curation, Writing – original draft, Visualization, Investigation, Writing – review & editing. **Nizar Al Bassam:** Conceptualization. **Amer Zayegh:** Software, Validation. **Sana Al Ghawi:** Writing – review & editing, Methodology.

## Declaration of Competing Interest

“This work was supported in part by Ministry of Higher Education Research and Innovation (MOHERI) formerly known as The Research council (TRC) of Oman under COVID-19 program Block Funding Agreement No TRC/CRP/MEC/COVID-19/20/09. The authors declare that they have no known competing financial interests or personal relationships which have or could be perceived to have influenced the work reported in this article.

## References

[1] Seshadri D.R., Davies E.V., Harlow E.R., Hsu J.J., Knighton S.C., Walker T.A., Voos J.E., Drummond C.K. (2020). Wearable sensors for COVID-19: a call to action to harness our digital infrastructure for remote patient monitoring and virtual assessments. Front. Digit. Health.

[2] Hussain S.A., Al Balushi A.S.A. (2020). A real time face emotion classification and recognition using deep learning model. J. Phys. Conf. Ser..

[3] Nooruddin S., Islam M., Sharna F. (2019). An IoT based device-type invariant fall detection system. Internet Things.

[4] El-Rashidy, N.; El-Sappagh, S.; Islam, S.M.R.; El-Bakry, H.M.; Abdelrazek, S. End-To-End Deep Learning Framework for Coronavirus (COVID-19) Detection and Monitoring. Electronics **2020**, 9, 1439. PP:1–25. 10.3390/electronics9091439.

[5] Hussain S.A., Hussain S.J., Hasan R., Mahmood S. (2021). Low-cost voice based braille script communication for teaching aid. J. Commun..

[6] Acho L., Vargas A.N., Vazquez G.P. (2020). Low cost, open-source mechanical ventilator with pulmonary monitoring for COVID-19 patients. Actuator, MDPI.

[7] Dagazany A.R., Stegagno P., Mankodiya K. (2018). WearableDL: wearable internet-of-things and deep learning for big data analytics—concept, literature, and future. Hindawi Mob. Inf. Syst..

[8] Qureshi F., Krishnan S. (2018). Wearable hardware design for the internet of medical things (IoMT). Sensors.

[9] Jahangir Alam Majumder A.K.M., ELsaadany Y.A., Young R., Ucci D.R. (2019). Energy efficient wearable smart IoT system to predict cardiac arrest. Hindawi Adv. Hum. Comput. Interact..

[10] Anto Arockia R.R., Lalitha R., Hariharan G., Lokesh N. (2020). Tracking the COVID zones through geo-fencing technique. Int. J. Pervasive Comput. Commun..

[11] Asri H., Mousannif H., Moatassime H.A. (2019). Reality mining and predictive analytics for building smart applications. J Big Data.

[12] Al Bassam N., Hussain S.A., Al Qaraghuli A., Khan J., Sumesh E.P., Lavanya V. (2021). IoT based wearable device to monitor the signs of quarantined remote patients of COVID-19. Inform Med Unlocked.

[13] Hussain S.J., Khan S., Hasan R., Hussain S.A., Mallick P., Balas V., Bhoi A., Chae G.S. (2020).

